# Procollagen C-Proteinase Enhancer 1 (PCPE-1) as a Plasma Marker of Muscle and Liver Fibrosis in Mice

**DOI:** 10.1371/journal.pone.0159606

**Published:** 2016-07-26

**Authors:** Eyal Hassoun, Mary Safrin, Hana Ziv, Sarah Pri-Chen, Efrat Kessler

**Affiliations:** Maurice and Gabriela Goldschleger Eye Research Institute, Tel-Aviv University Sackler Faculty of Medicine, Sheba Medical Center, Tel-Hashomer, 52621, Israel; University of Navarra School of Medicine and Center for Applied Medical Research (CIMA), SPAIN

## Abstract

Current non-invasive diagnostic methods of fibrosis are limited in their ability to identify early and intermediate stages of fibrosis and assess the efficacy of therapy. New biomarkers of fibrosis are therefore constantly sought for, leading us to evaluate procollagen C-proteinase enhancer 1 (PCPE-1), a fibrosis-related extracellular matrix glycoprotein, as a plasma marker of fibrosis. A sandwich ELISA that permitted accurate measurements of PCPE-1 concentrations in mouse plasma was established. Tissue fibrosis was assessed using histochemical, immunofluorescence, and immunoblotting analyses for type I collagen and PCPE-1. The normal plasma concentration of PCPE-1 in 6 weeks to 4 months old mice was ~200 ng/ml (189.5 ± 11.3 to 206.8 ± 13.8 ng/ml). PCPE-1 plasma concentrations in four and 8.5 months old *mdx* mice displaying fibrotic diaphragms increased 27 and 40% respectively relatively to age-matched control mice, an increase comparable to that of the N-propeptide of procollagen type III (PIIINP), a known blood marker of fibrosis. PCPE-1 plasma levels in mice with CCl_4_-induced liver fibrosis increased 34 to 50% relatively to respective controls and reflected the severity of the disease, namely increased gradually during the progression of fibrosis and went down to basal levels during recovery, in parallel to changes in the liver content of collagen I and PCPE-1. The results favor PCPE-1 as a potential new clinically valuable fibrosis biomarker.

## Introduction

Fibrosis is a non-physiological scarring process associated with excessive deposition of extracellular matrix (ECM), leading to impairment of organ function [1]. It can affect many organs and tissues, including liver, kidney, heart, lung, skin (hypertrophic scars, keloids) and skeletal muscles [1–3]. Various triggers can contribute to the development of fibrotic diseases, including inherited disorders, persistent infections, recurrent exposure to toxins, irritants or smoke, chronic autoimmune inflammation, myocardial infarction and hypertension [1–4]. A feature common to all fibrotic diseases is abnormal accumulation of collagen and other ECM components in the extracellular space that are produced by activated fibroblasts (myofibroblasts). This is evident for instance in left ventricular hypertrophy that results from chronic hypertension and may lead to heart failure [5] and liver fibrosis that is often caused by chronic hepatitis C virus infection or chronic alcohol abuse and may lead to cirrhosis and hepatocellular dysfunction [6].

Procollagen C-proteinase enhancer 1 (PCPE-1) is a connective tissue glycoprotein that increases the rate of release of the carboxyl-propeptide from fibrillar procollagens by procollagen C-proteinases (PCPs) [7–9], a reaction critical for the assembly of collagen fibrils. PCPE-1 (50/55 kDa for human and rodent PCPE-1, respectively) consists of two CUB (Complement-Uegf-Bone morphogenetic protein 1) domains that bind to the C-propeptide of types I and III procollagen and are required for enhancing activity, and a netrin-like (NTR) domain that mediates binding to heparin, heparan sulfate proteoglycans (e.g., syndecans) and fibronectin [10–12]. The enhancing activity of PCPE-1 appears to be restricted to fibrillar procollagens because it does not affect processing of any other PCPs substrate tested to date [13,14]. The tissue distribution of PCPE-1 overlaps that of collagen type I. It is abundant in tissues rich in collagen I such as tendon, bone, skin and cornea, expressed to a lower extent in tissues containing lower amounts of collagen I such as skeletal muscles, heart, and kidney, and is practically undetectable in organs producing no (or negligible amounts of) type I collagen such as brain and liver [8,10]. The expression of PCPE-1, like that of collagen type I, is up-regulated in organs undergoing fibrosis including liver [15,16], heart [17,18], skin (hypertrophic/keloid scars) [19] and cornea [20]. PCPE-1 expression in cultured fibroblasts is also coordinated with that of collagen I [15,16,18,20]. PCPE-1 is therefore recognized as an important regulator of collagen deposition and potential target for intervention with fibrosis [17,21]. Worth noting in this context, PCPE-1 is found in human sera [22–24], plasma [25] and cerebrospinal fluid [26,27] as well as rat plasma [16].

None-invasive diagnosis of fibrosis relies on imaging techniques and immunoassays of blood biomarkers [6, 28–30]. Blood markers used to evaluate liver fibrosis are classified as direct when they measure extracellular matrix components or indirect when they measure molecules released by the malfunctioning liver parenchyma [31]. Direct markers include (but are not limited to) the carboxyl propeptide of type I procollagen (PICP) and amino propeptide of type III procollagen (PIIINP) and both have also been used to evaluate cardiac fibrosis [31–35] although evidence that they actually reflect histologically proven myocardial fibrosis is still lacking [33,35]. Indirect markers of liver fibrosis include specific transaminases or molecules such as α2-macroglobulin, apolipoprotein A1, haptoglobin, γ-glutamyl transpeptidase (GGT) and bilirubin, which are components of a common assay of liver fibrosis known as Fibrotest [30,36]. Most of the currently available non-invasive diagnostic methods detect successfully advanced liver fibrosis (stages F2-F4) but they are not accurate enough in detecting early stages of the disease and discrimination between stages of advanced liver fibrosis [37,38]. Thus, at present, no clinical tools are available for monitoring progression of liver fibrosis [38]. Therefore, although invasive, liver biopsy, which permits accurate histologic assessment of disease severity, remains important in predicting prognosis and therapeutic outcome of liver disease [30,31,36]. Liver biopsy however is not free of limitations, in particular, risk of injury and sampling errors.

The limitations of the existing diagnostic methods of fibrosis highlight the importance of identifying and validating new biomarkers to monitor fibrosis. As a probable indicator of fibrosis, PCPE-1 could be useful in this regard. To explore this possibility, we developed a sandwich ELISA for mouse PCPE-1, which permitted determination of PCPE-1 concentrations in mouse plasma. We then examined its potential as a biomarker of fibrosis in two mouse models of fibrosis: *mdx* mice that mimic Duchenne Muscular Dystrophy (DMD), a genetic disorder caused by mutations in the dystrophin gene and associated with weakness in the diaphragm and myocardium, tissues most affected by fibrosis [3], and CCl_4_-induced liver fibrosis. We found that tissue fibrosis in both models was accompanied by a marked increase in the plasma concentrations of PCPE-1. Furthermore, in CCl_4_-induced liver fibrosis, changes in PCPE-1 plasma concentrations revealed both progression and regression of liver fibrosis, thus highlighting the potential of PCPE-1 as a new non-invasive biomarker for diagnosing and monitoring fibrosis.

## Materials and Methods

### Mice

Four and 8.5 months old *mdx* mice (males) were a kind gift of Dr. Mark Pines, The Hebrew University of Jerusalem, Israel. C57/BL/6 mice (WT; males) at the specified ages were purchased from Harlan, Israel. The study was approved by the Tel Aviv University Institutional animal care and use committee and performed in compliance with their guidelines.

### Proteins and antibodies

Mouse PCPE-1 (mPCPE-1) was purified from culture media of 3T6 mouse fibroblasts by ammonium sulfate fractionation, gel-filtration, lysyl-Sepharose chromatography and affinity chromatography on Sepharose coupled to the C-propeptide of type I procollagen as described [7]. To avoid spontaneous conversion of intact PCPE-1 to its CUB domains fragments of 34–36 kDa [7], protease inhibitors (10 mM N-ethylmaleimide and 1 mM phenylmethylsulfonyl fluoride) were added to the buffers used in the last step of purification (chromatography on the C-propeptide-Sepharose column). The purified PCPE-1 fraction (250 μg/ml in 0.05 M Tris-HCl, 0.15 M NaCl, 0.005 M CaCl_2_, 0.1% Brij 35, pH 7.5) appeared to be homogenous (S1 Fig) and was stored in aliquots at -80°C. Bovine serum albumin (BSA) was from Sigma (A7888). Rat monoclonal antibody against mPCPE-1 (MAB2239) and goat polyclonal antibody to mPCPE-1 (AF2239) were purchased from R&D. Alkaline phosphatase conjugated rabbit antibody to goat IgG and horseradish peroxidase (HRP) conjugated goat antibody to rabbit IgG were from Sigma. Cy2 conjugated goat antibody to rabbit IgG and Rhodamine conjugated donkey antibody to goat IgG were from Jackson Immunoresearch Ltd. Polyclonal antibody to type I collagen was from Acris Antibodies GmbH. Polyclonal rabbit antibody to the CUB1CUB2 fragment of mPCPE-1 was prepared as previously described [7]. A polyclonal rabbit antibody to the C-telopeptide of the α1 chain of human type I collagen (LF-67) was a kind gift of Dr. Larry Fisher [39]. A polyclonal rabbit antibody against *Pseudomonas aeruginosa* elastase was prepared as described [40]. An ELISA kit for determination of mouse procollagen III N-terminal propeptide (PIIINP) was purchased from USCN (cat. # E90573Mu).

### Mouse CCl_4_ liver fibrosis model

Induction of liver fibrosis by CCl_4_ was performed according to Perugorria et al [41]. Briefly, 6 weeks old C57/BL/6 mice weighing between 20 and 24 g were injected intraperitoneally with 80 μl of either CCl_4_ diluted 1:4 in olive oil or olive oil (vehicle control) twice a week for 6 weeks. Blood samples were collected (see below) before the first CCl_4_ administration, periodically during the injections period of 6 weeks, and at the indicated times after the last CCl_4_ injection.

### Preparation of plasma samples

Mice were anesthetized by isofluoran inhalation. Blood samples (100–200 μl) were collected from the peri-orbital sinus using a heparin coated capillary. Plasma was prepared by centrifugation (2,000 rpm, 10 min) and stored in aliquots at -80°C until use.

### Organ harvest

Mice were euthanized by CO_2_ inhalation. Diaphragms and livers were then removed from *mdx* and CCl_4_ treated mice, respectively as well as corresponding control mice. Each organ was divided into three portions that were manipulated as follows: (i) fixation in 4% paraformaldehyde for histochemistry (collagen staining); (ii) freezing in Optimal Cutting Temperature (OCT) solution for preparation of cryo-sections and immunofluorescence analysis; (iii) freezing at -80°C for protein extraction.

### Sandwich ELISA

A 96 well maxisorp plate (Nunc) was coated with monoclonal rat antibody to mPCPE-1 (50 μl; 5 μg/ml in 0.1 M carbonate buffer pH 9.2; overnight at 4°C). After washing with PBST (PBS plus 0.05% Tween-20), wells were blocked with 5% BSA/PBS (2 h, 30°C), washed with PBST, and 50 μl of either purified mPCPE-1 or plasma samples, both diluted in blocking buffer, were then added and incubated for 2 h at 30°C. Unbound PCPE-1 was removed by washing with PBST followed by addition of 50 μl goat anti mPCPE-1 polyclonal antibody (0.1 μg/ml in blocking buffer) and incubation for 60 min at 30°C. Wells were then washed with PBST followed by addition of 50 μl APA-conjugated rabbit antibody to goat IgG (diluted 1:2,000 in blocking buffer) and incubation for 45 min at 30°C. After washing with PBST, 100 μl of p-nitro-phenyl-phosphate (pNPP; Sigma; 1 mg/ml in 0.94 M diethanolamine-HCl pH 9.8) was added and the rate of release of p-nitrophenol was measured at 405 nm using an ELISA plate reader (Biotek, ELX808) heated at 30°C.

### Determination of coefficients of variability

To determine the inter-assay coefficient of variability, PCPE-1 concentration in plasma from a six weeks old C57/BL/6 mouse was determined repeatedly in ten independent experiments, each performed on separate days (samples were diluted 1:20 and 1:40 and each dilution was measured in duplicates. All of the values thus obtained were averaged; S1 Table; n = 4). The intra-assay coefficient of variability was determined in two different experiments, first for plasma samples from *mdx* mice and then for CCl_4_-treated-mice. In each instance, the test included 16 plasma samples (four *mdx* and four control mice at ages 4 and 8.5 months; eight CCl_4_-treated and eight control mice). Each plasma sample was diluted 1:20 and 1:40 and each dilution was analyzed either in triplicates (S2 Table; *mdx* model) or in duplicates (S3 Table; CCl_4_ liver fibrosis model) and the values obtained were averaged (n = 6 and 4 in S2 and S3 Tables, respectively).

### Immunoprecipitation

Protein-G-Sepharose beads (Sigma) were washed and blocked with 5% BSA/PBS (2h, 4°C, with constant shaking). After centrifugation, the pellet was re-suspended in the original volume of blocking buffer. Fifty μl samples of the resulting suspension were then incubated (4°C, overnight) with 3.3 μg of either goat anti mPCPE-1 IgG or rabbit IgG against *Pseudomonas aeruginosa* elastase (pseudolysin; unrelated antigen; negative control) in a total volume of 100 μl (adjusted by adding the appropriate volume of blocking buffer). Beads were then washed extensively with blocking buffer (to remove excess antibody) and suspended in 25 μl of blocking buffer. 110 μl of plasma from a 6 weeks old normal C57/BL/6 mouse (diluted 1:10 with blocking buffer) was added to each suspension and the beads were incubated (2 h, room temperature) to allow binding of plasma PCPE-1. Beads were then pelleted by centrifugation and the supernatants were stored at -80°C until use.

### Protein extraction from tissues

Tissue samples were weighed out (~ 100 mg each), cut into small pieces and homogenized (40 sec, on ice) in 0.5 ml PBS using a Polytron (Kinematica GmbH, Switzerland). Proteins were then solubilized by heating (100°C, 10 min) in Laemmli's sample buffer containing 1 mM PMSF (phenylmethylsulfonyl fluoride) and 10 mM EDTA but lacking bromophenol blue (total volume 1 ml). The heat denatured samples were centrifuged (14,000 rpm, 5 min) using an Eppendorf 5418 microfuge and the clear supernatants were collected and stored in aliquots at -20°C. Protein concentrations of tissue extracts were determined using a modification of the Lowry method adjusted for SDS-containing protein samples [42].

### Immunoblotting

Equal amounts of protein were applied on each lane, thus eliminating the need for a loading control. Proteins were resolved by SDS-PAGE in 6 and 10% gels for detection of the collagen α1(I) chain and PCPE-1, respectively, and were then electrotransferred to nitro-cellulose membranes (Bio-Rad) using 25 mM Tris-HCl, 192 mM glycine, 20% methanol, pH 8.3 as the transfer buffer. Membranes were blocked with 3% skimmed milk in 20 mM Tris-HCl, 137 mM NaCl, pH 7.6 (TBS; 2h, room temperature), washed in TBST (TBS plus 0.05%Tween-20), then incubated (4°C, overnight) with either rabbit IgG to the CUB1CUB2 fragment of mPCPE-1 or rabbit serum against human α1(I) C-telopeptide (LF-67) [39] diluted in 1% BSA in TBS containing 0.05% sodium azide (NaN_3_). After washing with TBST, each membrane was incubated with HRP-conjugated goat anti rabbit IgG and protein bands were visualized by ECL detection. Relative band intensities were determined by densitometry using TINA software version 2.07.

### Histochemistry

Tissue samples were fixed in 4% paraformaldehyde. After dehydration with increasing ethanol concentrations, tissue pieces were cleared in chloroform and embedded in paraffin. Four micron sections were then prepared using a Leica RM2155 microtome. After removal of the paraffin by heating at 60°C, sections were washed in toluene, rehydrated with decreasing ethanol concentrations, and were finally washed with water. Differential staining of collagenous and non-collagenous proteins was performed with 0.1% Fast green and 0.2% Sirius red in saturated picric acid [43]. Using this procedure, collagen is stained red and non-collagenous proteins are stained green (background).

### Immunofluorescence

Tissue samples were deep frozen in OCT (Tissue Tek, Japan). After freezing, 12 μm sections were prepared using a Leica CM3050S cryostat, fixed in 3.7% paraformaldehyde in PBS, washed in PBS containing 0.1% Tween-20 (PBST1), and blocked with 10% fetal calf serum (FCS) in PBST1 (60 min, room temperature). The sections were then incubated (4°C, overnight) with rabbit IgG against the CUB1CUB2 fragment and goat anti collagen type I antibody (diluted 1:100 and 1:1,000, respectively) in FCS/PBST1. After washing with PBST1, the sections were incubated with CY2 conjugated goat antibody to rabbit IgG and Rhodamine conjugated donkey antibody to goat IgG for detection of PCPE-1 and collagen type I, respectively. The sections were then washed again in PBST1, placed in Gel/Mount mounting medium (Golden Bridge International) and dried for 24 h at room temperature in the dark. Sections were examined at 580 nm (Rhodamine) and 488 nm (Cy2) using an Olympus BX51 fluorescence microscope. The stained areas in each panel were quantified by computerized image analysis using Olympus cellSens imaging software and expressed as the percent of the total panel's area.

### Statistical analysis

Differences between groups were analyzed for statistical significance using two-tailed independent t-tests. P values <0.05 were considered statistically significant.

## Results

### Sandwich ELISA for determination of plasma concentrations of mouse PCPE-1

**Fig 1:**
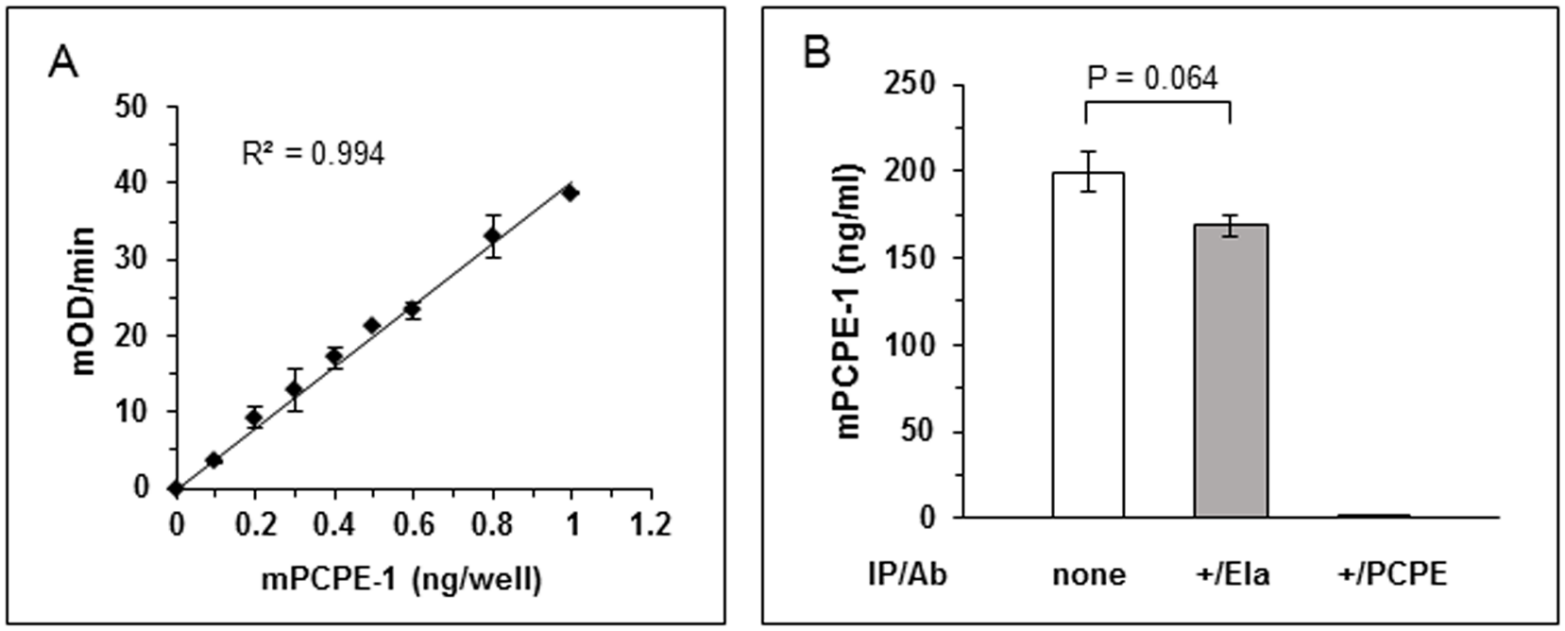
Sandwich ELISA for quantification of mPCPE-1. (A) Standard curve. Increasing amounts of purified mPCPE-1 were adsorbed to wells pre-coated with a rat monoclonal antibody to mPCPE-1. Bound mPCPE-1 was detected using a goat antibody to mPCPE-1 and quantified using an APA-conjugated rabbit anti goat IgG antibody. Each value represents mean ± standard deviation (SD); n = 2. mOD, Optical Density expressed in milli units; R^2^, coefficient of determination. (B) The ELISA is specific and permits determination of PCPE-1 concentration in mouse plasma. Plasma samples from a six weeks old C57/BL/6 mouse were subjected to IP with either goat antibody to mPCPE-1 (black) or a rabbit antibody to *Pseudomonas* elastase (negative control; grey). PCPE-1 concentrations in untreated plasma (white) and in plasma samples that underwent IP were determined using the sandwich ELISA. Values are mean ± SD (n = 4). Results are from a representative experiment out of two. **IP**, immunoprecipitation; **Ab**, antibody.

To quantify PCPE-1 in mouse plasma, we established a new sandwich ELISA employing rat monoclonal antibody against mouse PCPE-1 (mPCPE-1) to capture the antigen and a goat polyclonal antibody to mPCPE-1 for its detection. Quantification of bound mPCPE-1 was achieved using an alkaline phosphatase (APA) conjugated antibody to goat IgG and measuring the rate of release of p-nitrophenol (yellow chromophore) from the APA substrate, p-nitro-phenyl-phosphate. To determine the detection range and establish a standard/calibration curve, we assayed increasing amounts of purified mPCPE-1. A typical dose-dependent increase in absorbance at 405 nm was obtained that was linear in the range 0.1–1.0 ng mPCPE-1 (Fig 1A). The coefficient of determination (R^2^) was 0.994.

To test whether this ELISA procedure permits determination of PCPE-1 concentration in mouse plasma, we used a plasma sample (50 μl; diluted 1:20 and 1:40) from a six weeks old C57/BL/6 mouse. The plasma concentration of PCPE-1 was derived from a calibration curve similar to that shown in Fig 1A but attained on the day of the experiment. Based on this calibration curve, the average plasma concentration of PCPE-1 was found to be 199.15 ± 11.7 ng/ml (Fig 1B, two dilutions, each measured in duplicates).

To ascertain the specificity of the assay, PCPE-1 was removed from the plasma by immunoprecipitation (IP) followed by measurement of the amount of PCPE-1 remaining in the supernatant. As a negative control, we determined the amount of PCPE-1 in the plasma after IP with either beads harboring no antibody or beads coated with an antibody to *P*. *aeruginosa* elastase (unrelated antigen). Fig 1B shows that the amount of PCPE-1 in the plasma after IP with the elastase antibody was comparable to that of control plasma (168.16 ± 5.81 and 199.15 ± 11.7 ng/ml, respectively, which is equivalent to 84.4%; p = 0.064) whereas after IP with anti PCPE-1 antibody, the amount of PCPE-1 in the plasma was 2.14 ± 0.13 ng/ml, i.e., ~1% of the control (untreated plasma; p = 5x10^-9^). The amount of PCPE-1 remaining in the plasma after IP with beads harboring no antibody was 170.21 ± 2.93 ng/ml (not shown), essentially identical to that found after IP with the elastase antibody. Together, these results confirm that the newly developed ELISA permits determination of PCPE-1 concentrations in mouse plasma in a highly specific manner. The assay is also quite sensitive, permitting accurate measurements of 0.1–1 ng of mPCPE-1 (2–20 ng/ml). Furthermore, the assay is highly reproducible with an inter-assay coefficient of variability of 6.3% (S1 Table) and an intra-assay coefficient of variability between 5.15 and 8.5% depending on the experiment (S2 and S3 Tables), all below 10%—the maximal acceptable value.

### Plasma levels of PCPE-1 in *mdx* mice are elevated

**Fig 2:**
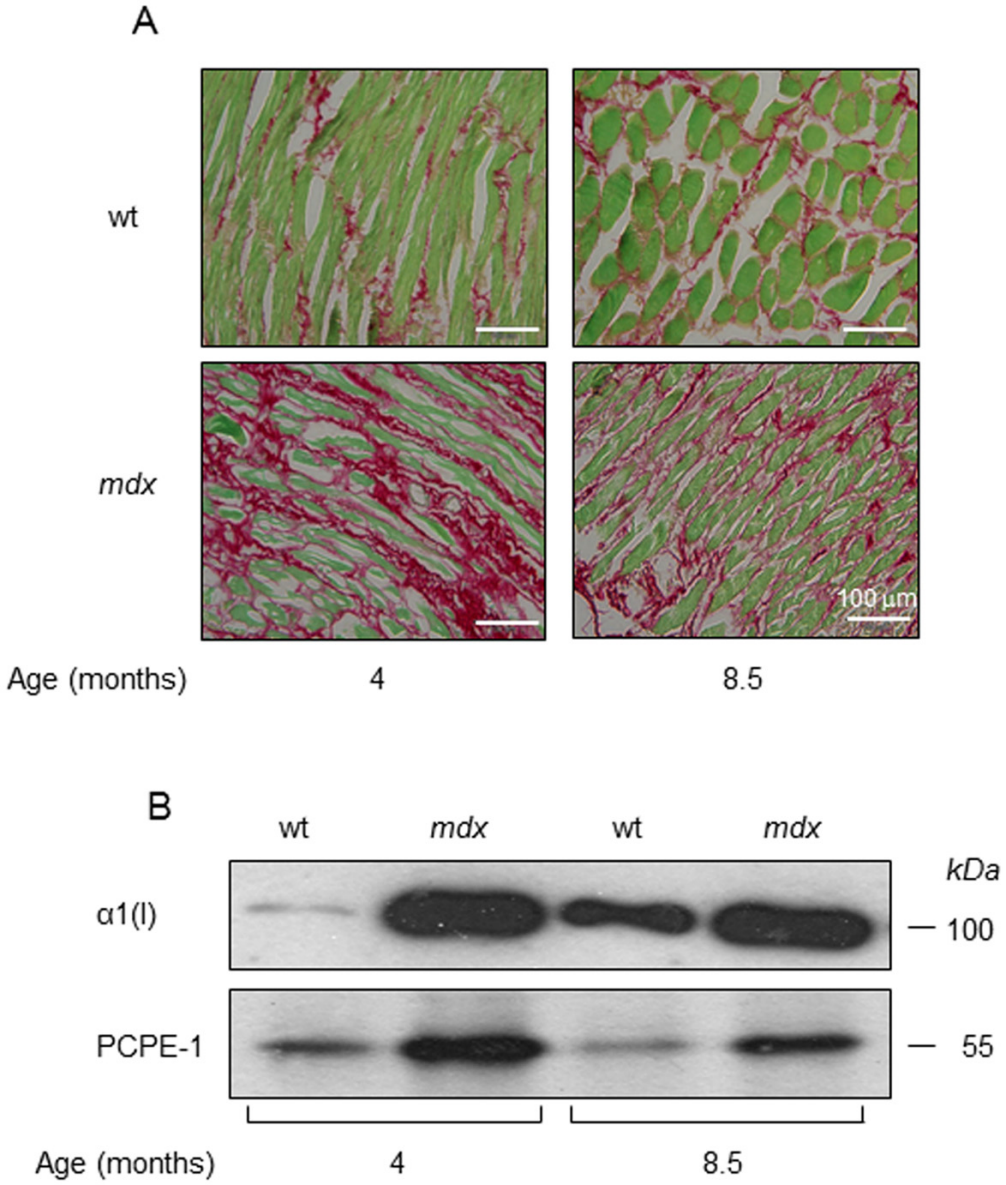
Diaphragms from *mdx* mice show increased collagen type I and PCPE-1 levels (A,B) and both are co-localized in the diaphragm tissue (C). **(A)** Sirius red staining of diaphragm sections from 4 and 8.5 months old *mdx* mice and age-matched C57/BL/6 (control, wt) (one mouse per group). **Red**, collagen; **Green**, non-collagenous proteins. (**B)** Immunoblot showing increased amounts of collagen type I and PCPE-1 in protein extracts of diaphragms from *mdx* mice relatively to corresponding wild type (wt) controls (one mouse per group). The amounts of protein applied on each lane were 1 and 25 μg for collagen type I and PCPE-1 detection, respectively. α1 (I), alpha 1 chain of type I collagen. *kDa*, kilo Dalton. (**C)** Immunofluorescence analysis for type I collagen and PCPE-1 in diaphragms from a four months old C57/BL/6 (wt) and an aged matched *mdx* mouse. Collagen type I (red) was detected using Rhodamine-conjugated secondary antibody and PCPE-1 (green) was visualized using a Cy2-conjugated secondary antibody. Light emission was examined at 488 and 580 nm for Rhodamine and Cy2, respectively. Results displayed in each panel are from a representative randomly selected mouse from each group.

Using the above ELISA, we tested whether the plasma concentrations of PCPE-1 in *mdx* mice reflect muscle fibrosis. Two groups of *mdx* mice (eight mice per group) at ages four and 8.5 months and two groups of age-matched wild type mice (strain C57/BL/6; control; eight mice per group) were studied. Occurrence of fibrosis in the diaphragm of a representative mouse from each group was assessed by Sirius red collagen staining. Fig 2A shows high collagen levels in diaphragms from four as well as 8.5 months old *mdx* mice relatively to corresponding wild type controls, indicating that the diaphragms of *mdx* mice are fibrotic. As evident from immunoblotting and immunofluorescence analyses (Fig 2B and 2C), the increase in collagen levels in these diaphragms was accompanied by increased PCPE-1 expression. The immunofluorescence analysis (Fig 2C) also revealed that collagen I and PCPE-1 were co-localized in the fibrotic tissue.

**Fig 3:**
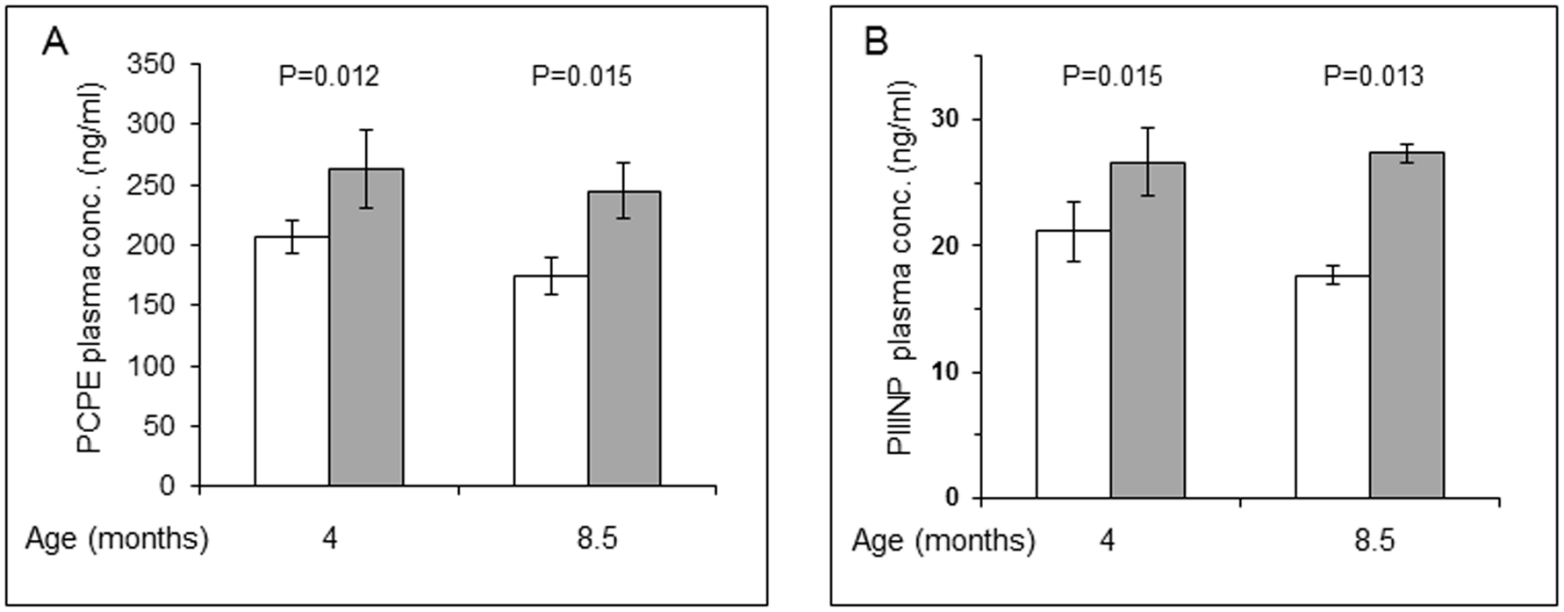
Plasma concentrations of PCPE-1 in *mdx* mice are higher than in the respective controls and the increase is comparable to that of PIIINP. **(A)** Plasma concentrations of PCPE-1 were determined using the current sandwich ELISA. Results are presented as the mean ± SD (n = 8). (**B)** Plasma concentrations of PIIINP were determined using a commercial kit. Each plasma sample was diluted 10 fold in blocking buffer and PIIINP concentrations were determined in duplicates. The results are presented as mean ± SD (n = 4 rather than 8 as in the case of PCPE-1 determination). This has to do with the high cost of the PIIINP kit. Nonetheless, and importantly, the results derived from four plasma samples were statistically valid.

Having shown that PCPE-1 expression is increased in diaphragms of *mdx* mice, we questioned whether PCPE-1 plasma concentrations in these mice are elevated. Fig 3A shows that the average plasma concentrations in four and 8.5 months old *mdx* mice were higher than those of the control. At four months of age, the average plasma concentration of PCPE-1 in the control group was 206.8 ± 13.8 ng/ml, practically identical to that of a six weeks old control mouse (199.15 ± 11.7 ng/ml) whereas the average PCPE-1 plasma concentration in four months old *mdx* mice was 262.9 ± 32.6 ng/ml, 27% higher than the control (n = 8/group; p = 0.012; statistically significant). The average plasma concentration in 8.5 months old control mice was 174.3 ± 15.5 ng/ml while in *mdx* mice it was 244.8 ± 23.0 ng/ml, namely, 40% higher than the control (n = 8/group; p = 0.015; statistically significant). Worth noting, the average plasma concentration of PCPE-1 in 8.5 months old control mice (174.3 ± 15.5 ng/ml) was somewhat lower than that of 4 months old control mice (206.8 ± 13.8 ng/ml) and so were the values in *mdx* mice of both ages (262.9 ± 32.6 and 244.8 ± 23.0 ng/ml in 4 and 8.5 months old mice, respectively). This difference may reflect a slower turnover rate of collagen and PCPE-1 at old age. Nevertheless, the results indicate that PCPE-1 plasma concentrations in *mdx* mice displaying fibrotic diaphragms are elevated, supporting the potential of PCPE-1 as a plasma marker of fibrosis.

For further evaluation of PCPE-1 as a new plasma marker of fibrosis, we compared PCPE-1 to a well-established marker of fibrosis, the N-propeptide of type III procollagen (PIIINP). Using a commercial ELISA kit for PIIINP, we measured its concentration in plasma samples from four control and four *mdx* mice that were each randomly selected. The results (Fig 3B) show that PCPE-1 was comparable to PIIINP in its ability to reflect muscle fibrosis in *mdx* mice. At four months of age, the average plasma concentration of PIIINP in *mdx* mice (26.6 ± 2.7 ng/ml) was ~26% higher than that of the control group (21.1 ± 2.4 ng/ml; n = 4; p = 0.015), essentially the same increase as that found for PCPE-1 (27%). At 8.5 months of age, the average plasma concentration of PIIINP in *mdx* mice (27.4 ± 0.7 ng/ml; n = 4) was about 55% higher than that of the control group (17.7 ± 0.8 ng/ml; p = 0.013). Since the extent of increase in PCPE-1 and PIIINP plasma levels at both ages was either identical or in the same range, we conclude that PCPE-1 is comparable to PIIINP in its ability to reflect tissue fibrosis in *mdx* mice.

### Plasma levels of PCPE-1 are elevated in liver fibrosis

**Fig 4:**
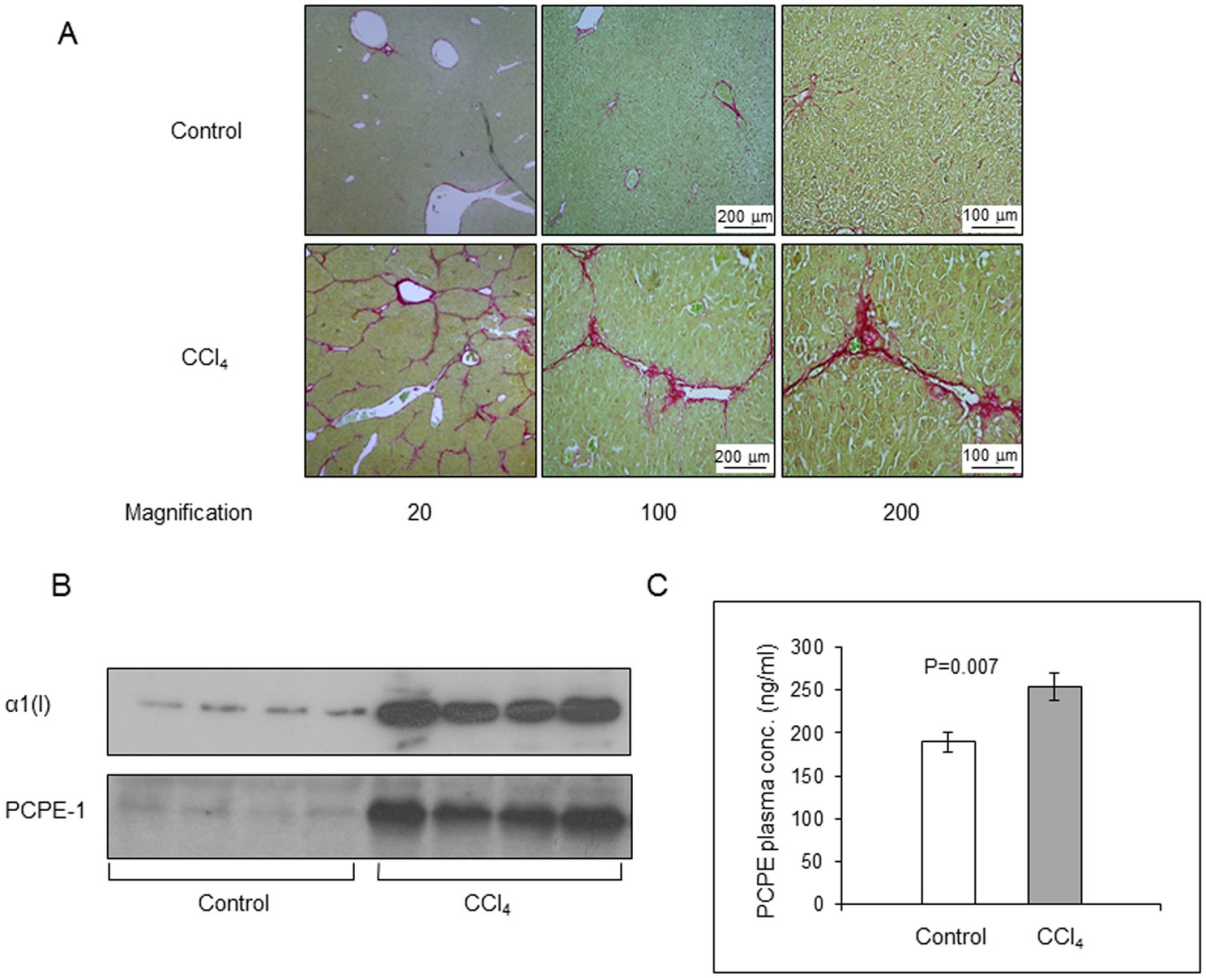
CCl_4_-induced liver fibrosis in mice is accompanied by increased PCPE-1 plasma concentration. CCl_4_ was given to C57/BL/6 mice for 6 weeks as detailed in Methods. **(A)** Sirius red staining of liver sections from randomly selected control and CCl_4_-treated mice shows increased collagen expression in the liver of the CCl_4_-treated mouse. **Red**, collagen; **Green**, non-collagenous proteins. **(B)** Immunoblot showing increased amounts of collagen type I and PCPE-1 in livers from CCl_4_-treated mice as compared to control mice. Each lane represents liver extract from a single randomly selected mouse of the indicated group. The amount of protein applied on each lane was 5 and 24 μg for collagen type I and PCPE-1 detection, respectively. Samples from 4 (out of 8) mice were analyzed per group. α1 (I), alpha 1 chain of type I collagen. (**C)** Plasma concentrations of PCPE-1 in CCl_4_-treated mice are significantly higher than those of control mice (n = 8).

Liver fibrosis in mice was induced by intraperitoneal injection of CCl_4_ twice a week for 6 weeks. Sirius red staining of liver sections from a representative CCl_4_-treated mouse revealed a marked increase of collagen type I as compared to control (Fig 4A). Immunoblotting showed that the increase in collagen type I content in liver extracts from four (randomly selected) CCl_4_-treated mice was associated with a similar increase in PCPE-1 content (Fig 4B). Densitometry of band intensities revealed that the content of both collagen I and PCPE-1 in these liver extracts increased about 9 fold as compared to control. Fig 4C shows that the average plasma concentration of PCPE-1 in the control group was 189.5 ± 11.3 ng/ml whereas that of the CCl_4_-treated mice was 254.1 ± 15.4 ng/ml, i.e., about 34% higher than the control (n = 8; p = 0.007). Importantly, the average plasma concentration of PCPE-1 in control mice was essentially the same as that found in the preceding experiments for 6 weeks and 4 months old control mice (199.15 ± 11.7 and 206.8 ± 13.8 ng/ml, respectively). This established the value of ~200 ng/ml as the base line plasma concentration of PCPE-1 in young mice.

### Plasma levels of PCPE-1 reflect the progression of liver fibrosis in CCl_4_-treated mice

**Fig 5:**
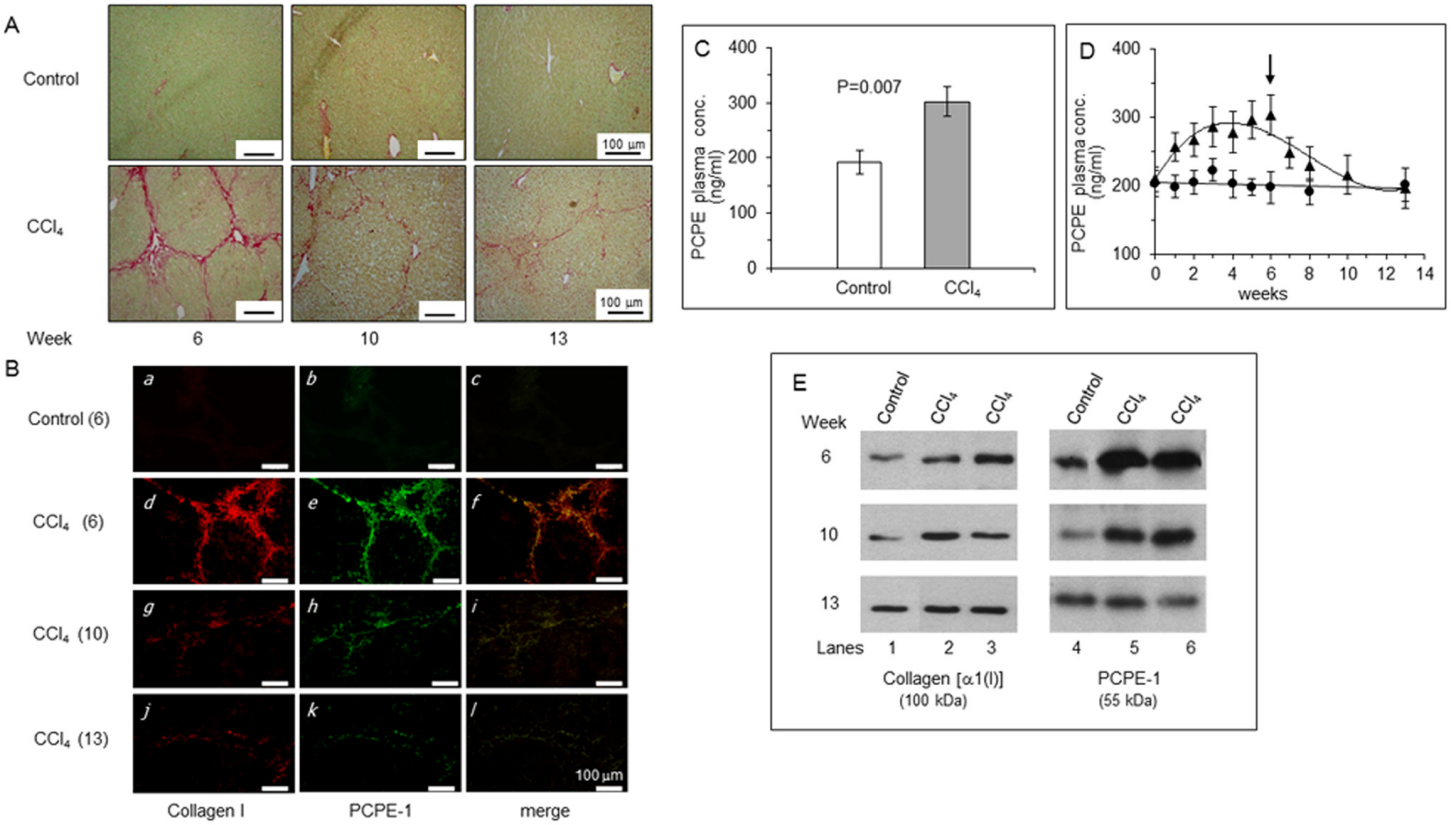
Plasma concentrations of PCPE-1 reflect the progression of liver fibrosis in CCl_4_-treated mice. C57/BL/6 mice were given CCl_4_ for 6 weeks and then remained untreated for 7 additional weeks. Liver fibrosis was assessed at the indicated times by Sirius red staining, immunoblotting, and immunofluorescence analyses. Plasma concentrations of PCPE-1 were determined by the sandwich ELISA at the indicated time points. **(A**) Sirius Red staining of liver sections from mice euthanized at weeks 6, 10 and 13. Each panel shows results for a single randomly selected mouse from the indicated group. **Red**, collagen; **Green**, non-collagenous proteins. **(B**)Immunofluorescence for type I collagen and PCPE-1 in liver sections from CCl_4_-treated and control mice at different times (given in brackets as the number of weeks). Each panel displays results for a single randomly selected mouse from the indicated group. Collagen type I (red) and PCPE-1 (green) were detected using Rhodamine- and Cy2-conjugated secondary antibodies at 488 and 580 nm, respectively. **(C)** Plasma concentrations of PCPE-1 after 6 weeks of CCl_4_ administration are significantly higher than those of control mice (n = 12). **(D**) Plasma concentrations of PCPE-1 in CCl_4_-treated (▲) and control (●) mice as a function of time. **Arrow,** termination of CCl_4_ treatment. Results are presented as mean ± SD. (n at weeks 0 to 6 = 12; n at weeks 7–10 = 9; n at week 13 = 6). **(E)** Immunoblot evaluating collagen I and PCPE-1 content in liver extracts from CCl_4_-treated and control mice at weeks 6, 10 and 13. Samples from a single control mouse and two CCl_4_-treated mice (all randomly selected) were analyzed. The amounts of protein applied on each lane were 4 and 36 μg for collagen and PCPE-1 detection, respectively.

To examine whether the plasma levels of PCPE-1 reflect the severity of liver fibrosis, we followed changes in the plasma concentrations of PCPE-1 and the degree of liver fibrosis as a function of time during (and after termination of) CCl_4_ treatment. Blood was withdrawn from CCl_4_-treated and control mice once a week in the course of CCl_4_ treatment (six weeks; n/group = 12) as well as one, two, four and seven weeks after termination of CCl_4_ treatment (recovery period). At week six (after bleeding), three mice from each group were euthanized for evaluation of liver fibrosis. The remaining mice (from now on, untreated) were bled at weeks seven, eight and ten, at which time, three additional mice from each group were euthanized, thus leaving six animals per group for determination of PCPE-1 plasma concentration and evaluation of liver fibrosis once again at week thirteen (seven weeks of recovery). Fibrosis was assessed at the (above) indicated time points by Sirius red collagen staining as well as immunoblotting and immunofluorescence analyses for collagen and PCPE-1. The results of Sirius red staining are presented in Fig 5A, showing that at week six (end of CCl_4_ treatment), considerable increase in collagen content was found in the liver of a representative CCl_4_-treated mouse as compared to control, which was greatly reduced at weeks ten and thirteen (recovery period) Immunofluorescence analysis (Fig 5B) revealed that collagen I and PCPE-1were both highly expressed at week six (end of CCl_4_ treatment) relatively to control (compare panels *d* and *e* to panels *a* and *b*, respectively) and that expression of both was greatly reduced at weeks ten (four weeks of recovery; panels *g*, *h*) and thirteen (seven weeks of recovery; panels *j*, *k*), reaching baseline levels at week thirteen. Essentially the same pattern was revealed by immunoblotting (Fig 5E) and as in the case of fibrotic diaphragms from *mdx* mice (Fig 2C), collagen I and PCPE-1 were co-localized in fibrotic liver tissue (Fig 5B, panel *f*).

**Fig 6:**
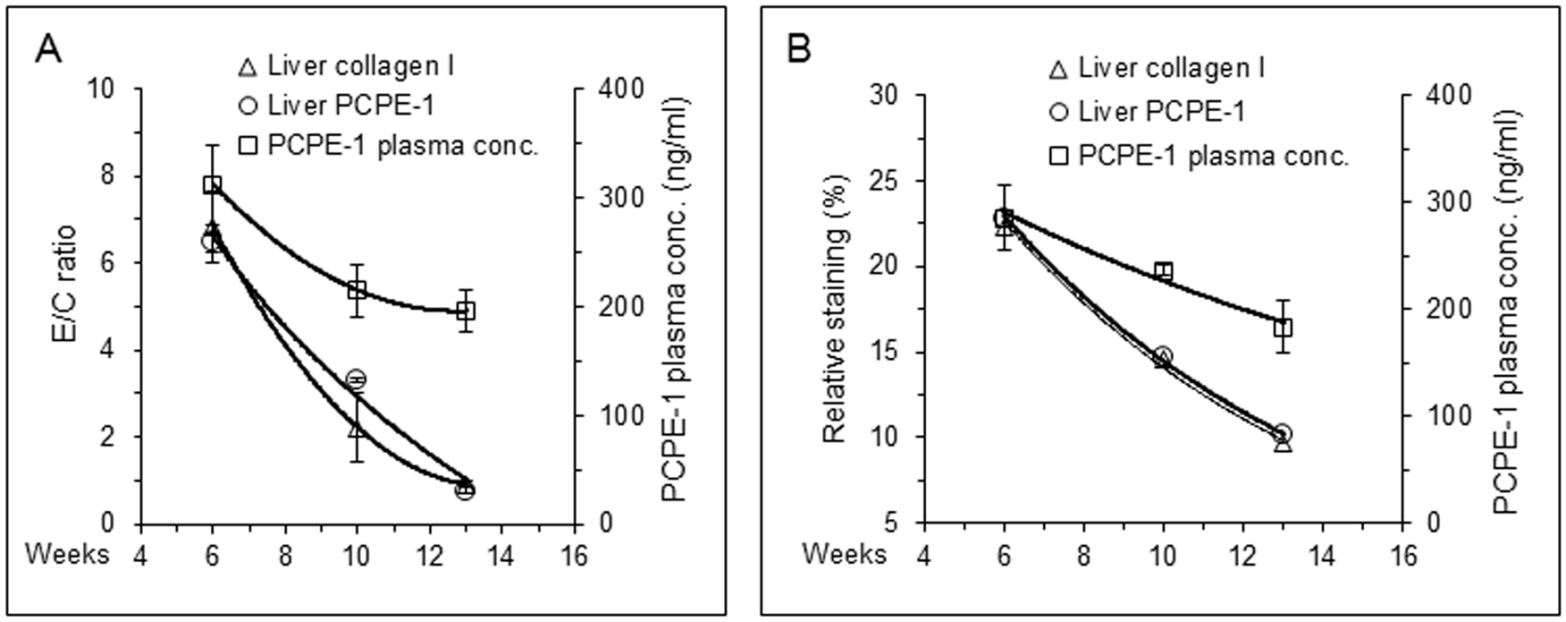
Plasma concentrations of PCPE-1 decline during liver recovery in parallel to the decline of type I collagen and PCPE-1 liver content. **(A)** The relative amounts of collagen type I were determined by densitometry of the respective α1(I) protein bands in the immunoblot shown in Fig 5E while those of PCPE-1 were derived from a similar blot (not shown) in which the total amount of protein applied on each lane was 12 μg (instead of 36 μg, as was used in Fig 5E), which was required to achieve linearity of band intensity in densitometry. E/C, ratio of absorbance units obtained for CCl_4_-treated (E) and control (C) mice, respectively. Each data point represents the mean of two E/C ratios calculated by dividing the relative band intensities from each of the two CCl_4_-treated mice (lanes 2 and 3 for the α1(I) collagen chain; lanes 5 and 6 for PCPE-1) by the relevant band intensities obtained for the respective control mouse (lanes 1 and 4 for α1(I) and PCPE-1, respectively). The values used to draw the graphs are mean ± SD (n = 2). Values of PCPE-1 plasma concentrations represent the mean ± SD obtained from 12, 9 and 6 CCl_4_-treated mice at weeks 6, 10 and 13, respectively. **(B)** The relative amounts of PCPE-1 and collagen type I in the liver were derived from the immunofluorescence analysis shown in Fig 5B. Values represent relative staining, expressed as the percentage of stained area out of the total area in each panel. Values correspond to data derived for a single representative mouse per group at the specified time point. Plasma concentrations of PCPE-1 are those obtained for the respective mice. Each value is the mean ± SD of four independent measurements (two dilutions, each measured in duplicates).

Once the degree of liver fibrosis was evaluated as above, we went on to determine the respective PCPE-1 plasma concentrations, asking to what extent the plasma levels of PCPE-1 were relevant to the severity of liver fibrosis. Fig 5C compares the average plasma concentrations of PCPE-1 in control and CCl_4_-treated mice at week six (peak of fibrosis) and Fig 5D displays changes in PCPE-1 plasma concentrations in these groups as a function of time. At week six, the average plasma concentration of PCPE-1 in control mice (n = 12) was 192 ± 21.2 ng/ml, which is consistent with data obtained in the preceding experiments, whereas that of the CCl_4_-treated mice (n = 12) was 302 ± 26.1 ng/ml, 57% higher (p = 0.007). Furthermore, the plasma concentrations of PCPE-1 in CCl_4_-treated mice increased gradually during the first six weeks of the experiment, leveling up between weeks four and six, and then, decreased gradually, reaching the basal level of control mice at week thirteen (seven weeks of recovery; Fig 5D). The plasma concentrations of PCPE-1 in the control group remained practically unchanged throughout the experiment. The relative amounts of PCPE-1 and collagen I in the liver at weeks six, ten and thirteen were quantified by measuring their respective band intensities in the immunoblot shown in Fig 5E and their relative staining in the immunofluorescence analysis shown in Fig 5B*d*, 5B*e*, 5B*g*, 5B*h*, 5B*j* and 5B*k*. Because of technical reasons, in the immunoblotting experiment we present data from a single control mouse (instead of two) and two CCl_4_-treated mice at each time point, all randomly selected. The intensity of the protein bands was determined by densitometry and the results are presented as a ratio E/C in which E and C stand for CCl_4_-treated and control mice, respectively. The ratio at each time point was calculated using the individual values obtained for each CCl_4_-treated mouse divided by the value obtained for the respective control mouse. The resulting E/C values at each time point were averaged and are presented in Fig 6A as mean ± SD (n = 2). As seen, the curves obtained for the relative levels of PCPE-1 and collagen type I in the liver are almost identical, indicating that changes in the liver content of both proteins occurred in parallel. Furthermore, changes in the plasma concentrations of PCPE-1 in these mice paralleled the changes in the liver content of collagen I and PCPE-1, namely, reflected the degree of liver fibrosis, thus supporting the thought that plasma levels of PCPE-1 can shed light on the severity of fibrosis. Similar results were derived from the immunofluorescence analysis shown in Fig 5B. In this instance, the values obtained for the relative staining of collagen I and PCPE-1 at weeks six, ten, and thirteen (expressed as percent of stained area relatively to the entire area of each panel) were practically identical: 22.3, 14.6 and 9.7% for collagen I (Fig 5B*d*, 5B*g* and 5B*j*) and 22.8, 14.8 and 10.1% for PCPE-1, respectively (Fig 5B*e*, 5B*h* and 5B*k*). The curves depicting relative staining of PCPE-1 and collagen I as a function of time (Fig 6B) were therefore superimposed, revealing once again that changes in collagen I and PCPE-1 expression at each stage of the experiment were coordinated. Likewise, changes in the plasma concentrations of PCPE-1 paralleled changes in the liver content of collagen I and PCPE-1 (Fig 6B), reinforcing the capability of PCPE-1 as a plasma marker of liver fibrosis.

## Discussion

The linkage between excessive collagen deposition and up-regulation of PCPE-1 in fibrosis is well established [15–20] and so is the occurrence of PCPE-1 in sera [22–25]. Moreover, PCOLCE, the gene encoding PCPE-1, has been identified as one of the top predictive genes and most differentially expressed gene in experimental liver fibrosis in rat [16]. Additionally, the plasma levels of PCPE-1 in rats treated with fibrogenic compounds were found to be higher than in rats treated with non-fibrogenic compounds [16]. PCPE-1 has also been identified as a serum marker of bone disorders in humans, in particular, Paget's disease and osteoporosis [24]. Together, the above findings point at PCPE-1 as a promising plasma marker of fibrosis, a possibility tested here for the first time in a systematic manner.

An assay for determination of PCPE-1 plasma concentrations was indispensable for the study. Commercial ELISA kits to measure human and rodent PCPE-1 concentrations are available but these were excluded because of their high cost. PCPE-1 concentrations in sera can also be measured by isoelectric focusing combined with immunoblotting and detection with a bio-imaging camera [23,24]. This method however is laborious and requires specialized costly equipment. In addition, the precision of this assay is borderline (mean intra- and inter-assay CVs of 15 and 20%, respectively) [24]. To overcome the above limitations, we decided to develop a new ELISA for mPCPE-1. Besides its simplicity, the assay we established is sensitive, specific, accurate and reproducible (inter- and intra-assay CV <10%) and permits determination of PCPE-1 concentrations in mouse plasma (Fig 1), thereby meeting the criteria mandatory for such assays. In addition, the ELISA we describe utilizes commercially available antibodies and is inexpensive. Thus, it can be adapted by other laboratories and is offered as a new research tool in the field.

We chose *mdx* mice as an initial experimental model because fibrosis of skeletal muscles in these mice occurs naturally and predictably [3,44]. The aim here was primarily to validate that plasma concentrations of PCPE-1 can reveal tissue fibrosis. Before assaying the plasma concentrations of PCPE-1 in these mice, it was critical to ascertain that the diaphragms of the *mdx* mice studied were fibrotic. This was achieved by Sirius red collagen staining, immunoblotting and immunofluorescence analyses, all of which revealed increased collagen deposition in diaphragms from 4 and 8.5 months old *mdx* mice (Fig 2). Furthermore, consistent with previous reports by us [15,17,21] and others [16,20], the increase in collagen expression was accompanied by increased PCPE-1 expression. Most notably, our immunofluorescence analysis showed for the first time that PCPE-1 and collagen type I are co-localized in the tissue (Fig 2C), further highlighting the relevance of PCPE-1 to collagen maturation.

The average PCPE-1 plasma concentration in young mice (~200 ng/ml) was somewhat higher than in old mice (~174 ng/ml) however both values are comparable to that found for human sera (~300 ng/ml) [24]. Surprisingly, the reported concentration of PCPE-1 in rat plasma (~1.5 ng/ml) [16] was two orders of magnitude lower than those found for human and mouse. The reason for this vast difference is not clear. Theoretically, though unlikely, this could result from a considerably lower degree of PCPE-1 leakage from the ECM into the blood in the rat. A more likely explanation may lie in the assay method. Rat PCPE-1 was determined using a commercial ELISA kit [16] in which an unknown PCPE-1 preparation was used for calibration. Inappropriate handling of the PCPE-1 standard solution by investigators who are not familiar with the biochemical properties of PCPE-1 could also account for the difference. Regardless and importantly, using the *mdx* mouse model we demonstrate here for the first time that PCPE-1 plasma concentrations are elevated in mice experiencing fibrosis.

An important aspect in the evaluation of PCPE-1 as a new biomarker of fibrosis was a comparison to an established marker of collagen biosynthesis. Since PCPE-1 is involved in C-terminal procollagen processing, from a mechanistic point of view, the natural choice for comparison should have been the carboxyl propeptide of type I procollagen (PICP). We selected PIIINP instead because it is probably the most evaluated marker in the field of fibrosis and the preferred diagnostic marker in liver fibrosis [38]. Using the *mdx* model, we showed that PCPE-1 is comparable to PIIINP (Fig 3), thus supporting the likelihood that PCPE-1 may emerge as a new biomarker of fibrosis.

Interestingly, the plasma levels of PCPE-1 as well as PIIINP in control 8.5 months old mice (174±15 and 17.7±0.8 ng/ml, respectively) were somewhat lower than those found for four months old control mice (206±13.8 and 21.1±2.4 ng/ml, respectively (Fig 3). This finding was puzzling because aging is often accompanied by increased organ fibrosis. A possible explanation could come from the findings that collagen biosynthesis in skeletal muscle from old animals is lower than in young animals [45] and that collagen synthesis is reduced in aging human dermal fibroblasts [46]. Our finding that plasma levels of PCPE-1 and PIIINP are slightly lower in old animals than in young ones is in agreement with the above documentation of decreased collagen biosynthesis in old age.

To further evaluate PCPE-1 as a fibrosis biomarker, we switched to a liver fibrosis model, which was selected for the following reasons. First, liver fibrosis is a common pathological condition representing one of the leading causes of death worldwide [47]. Secondly, new non-invasive diagnostic tools to monitor the severity of liver fibrosis are constantly sought for [31,32,37,48]. Thirdly, while the progression of muscle fibrosis in *mdx* mice is slow and cannot be modified experimentally, in experimental liver fibrosis, the course and severity of the disease can be manipulated. Also, the time frame is relatively short (weeks) and the organ involved is large, thus changes in PCPE-1 plasma levels are expected to be better pronounced. We selected the CCl_4_ liver fibrosis model because in this model substantial fibrosis is evident in 4–6 weeks and increased collagen production can be detected as early as 5 days after initiation of the experiment [16,41]. Under the experimental conditions we used, considerable liver fibrosis (increased amounts of collagen I and PCPE-1 in the liver) was evident after 6 weeks of CCl_4_ administration (Fig 4A and 4B; Fig 5A, 5B and 5E), which was accompanied by about 34% increase in the plasma levels of PCPE-1 (Fig 4C), thus corroborating the results gained using *mdx* mice as a model. Because most blood markers of liver fibrosis do not discriminate between early and intermediate stages of the disease [29,30,48], we examined to what extent PCPE-1 plasma concentrations of PCPE-1 can do so. The results were promising. PCPE-1 plasma levels clearly reflected the progression and regression of liver fibrosis, increasing in parallel to the progression of the disease and decreasing in parallel to its regression (Figs 5 and 6). The finding that PCPE-1 plasma concentration increased significantly after no more than two injections of CCl_4_ (day seven; Fig 5D) and decreased gradually during recovery (Figs 5D and 6) suggests that PCPE-1 plasma levels can reveal early fibrosis and discriminate between early, intermediate and severe fibrosis. In addition to the association between PCPE-1 plasma levels and tissue levels of collagen type I and PCPE-1, the data presented in Fig 6 highlight the tight linkage between collagen type I and PCPE-1 production.

A comparison of PCPE-1 to PIIINP in the CCl_4_ liver fibrosis model, in particular, during progression and regression of fibrosis, as was done for the *mdx* model could have been highly instructive and valuable. This however was not feasible because of the high cost of the commercial PIIINP kit required to assay so many plasma samples. Studies to illuminate this point are warranted.

While our data favor PCPE-1 as a useful biomarker of fibrosis, it is probably unlikely that a single bio-molecule, including PCPE-1, would be sufficient as an indicator of early fibrosis. A more promising direction could be utilization of a number of fibrosis-related biomolecules that in combination would facilitate detection of early fibrosis and monitoring the outcome of therapy. PCPE-1 could be a constituent of such combinations comprising previously established fibrosis markers (e.g., PICP, PIIINP) and/or recently suggested candidates, including fibronectin, connective tissue growth factor, lysyl oxidase, and lysyl oxidase-like protein-2 [48]. Such combinations could offer in the future highly effective methods for staging fibrosis. The contribution of PCPE-1 in this regard warrants further investigations in experimental models of fibrosis as well as clinically relevant situations.

## Supporting Information

S1 Fig SDS-PAGE analysis of the purified mPCPE-1 used as a standard for calculating PCPE-1 concentrations in the ELISA. (TIF)

S1 Table Determination of inter-assay coefficient of variability for the mPCPE-1 sandwich ELISA. (DOCX)

S2 Table Determination of intra-assay coefficient of variability for the mPCPE-1 sandwich ELISA—the *mdx* model. (DOCX)

S3 Table Determination of intra-assay coefficient of variability for the mPCPE-1 sandwich ELISA—the CCl_4_ liver fibrosis model. (DOCX)
