# Correction: Procollagen C-Proteinase Enhancer 1 (PCPE-1) as a Plasma Marker of Muscle and Liver Fibrosis in Mice

**DOI:** 10.1371/journal.pone.0162747

**Published:** 2016-09-06

**Authors:** Eyal Hassoun, Mary Safrin, Hana Ziv, Sarah Pri-Chen, Efrat Kessler

Fig 2 is incomplete as Panel C is missing. The authors have provided the complete Fig 2 here.

**Fig 2:**
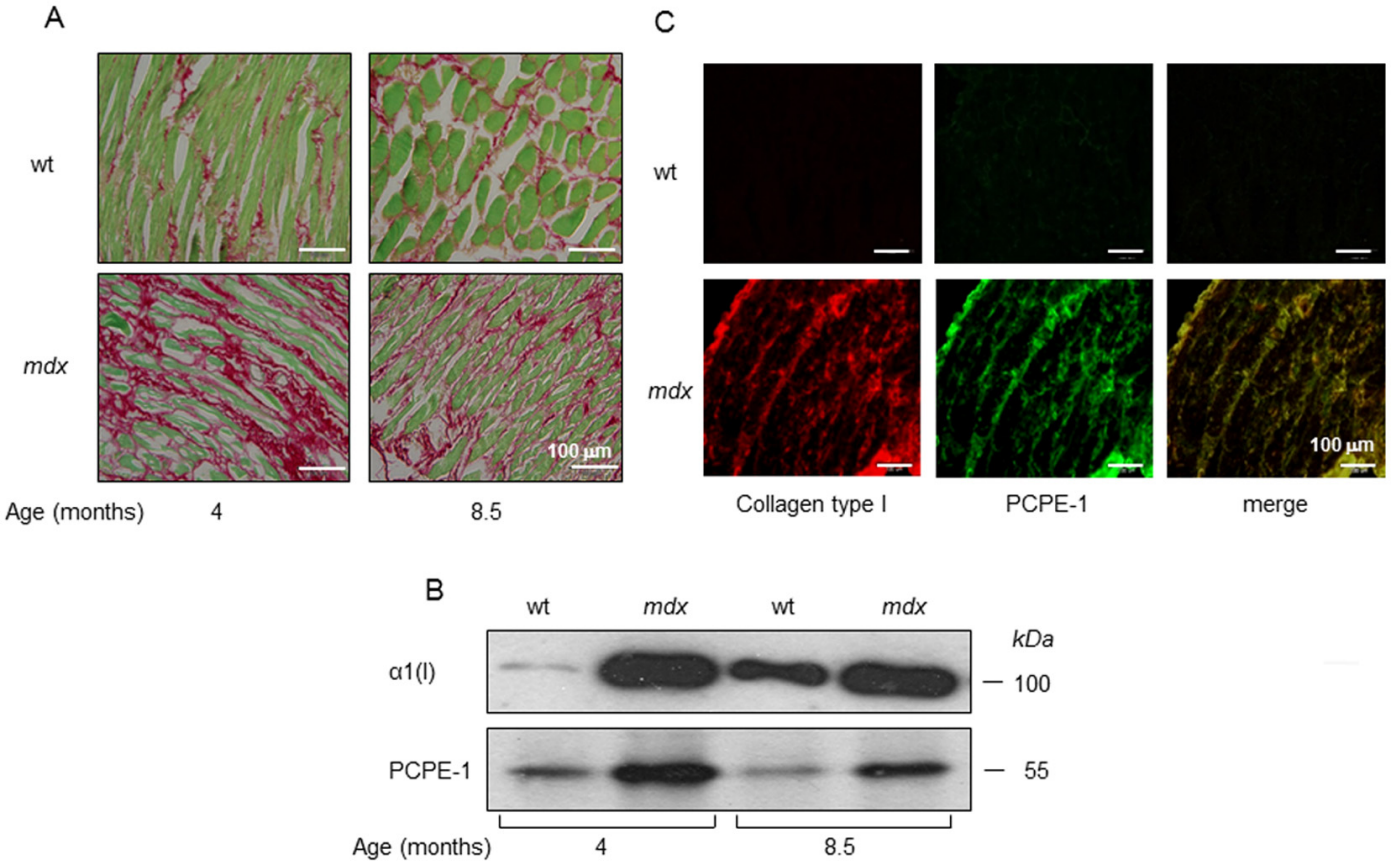
Diaphragms from *mdx* mice show increased collagen type I and PCPE-1 levels (A,B) and both are co-localized in the diaphragm tissue (C). **(A)** Sirius red staining of diaphragm sections from 4 and 8.5 months old *mdx* mice and age-matched C57/BL/6 (control, wt) (one mouse per group). **Red**, collagen; **Green**, non-collagenous proteins. **(B)** Immunoblot showing increased amounts of collagen type I and PCPE-1 in protein extracts of diaphragms from *mdx* mice relatively to corresponding wild type (wt) controls (one mouse per group). The amounts of protein applied on each lane were 1 and 25 μg for collagen type I and PCPE-1 detection, respectively. α1 (I), alpha 1 chain of type I collagen. *kDa*, kilo Dalton. **(C)** Immunofluorescence analysis for type I collagen and PCPE-1 in diaphragms from a four months old C57/BL/6 (wt) and an aged matched *mdx* mouse. Collagen type I (red) was detected using Rhodamine-conjugated secondary antibody and PCPE-1 (green) was visualized using a Cy2-conjugated secondary antibody. Light emission was examined at 488 and 580 nm for Rhodamine and Cy2, respectively. Results displayed in each panel are from a representative randomly selected mouse from each group.
