# Supplementary figures and images for: Procollagen C-Proteinase Enhancer 1 (PCPE-1) as a Plasma Marker of Muscle and Liver Fibrosis in Mice
Source: PLoS One. 2016 Jul 26;11(7):e0159606. doi: 10.1371/journal.pone.0159606 (PMC4961444; doi:10.1371/journal.pone.0159606)

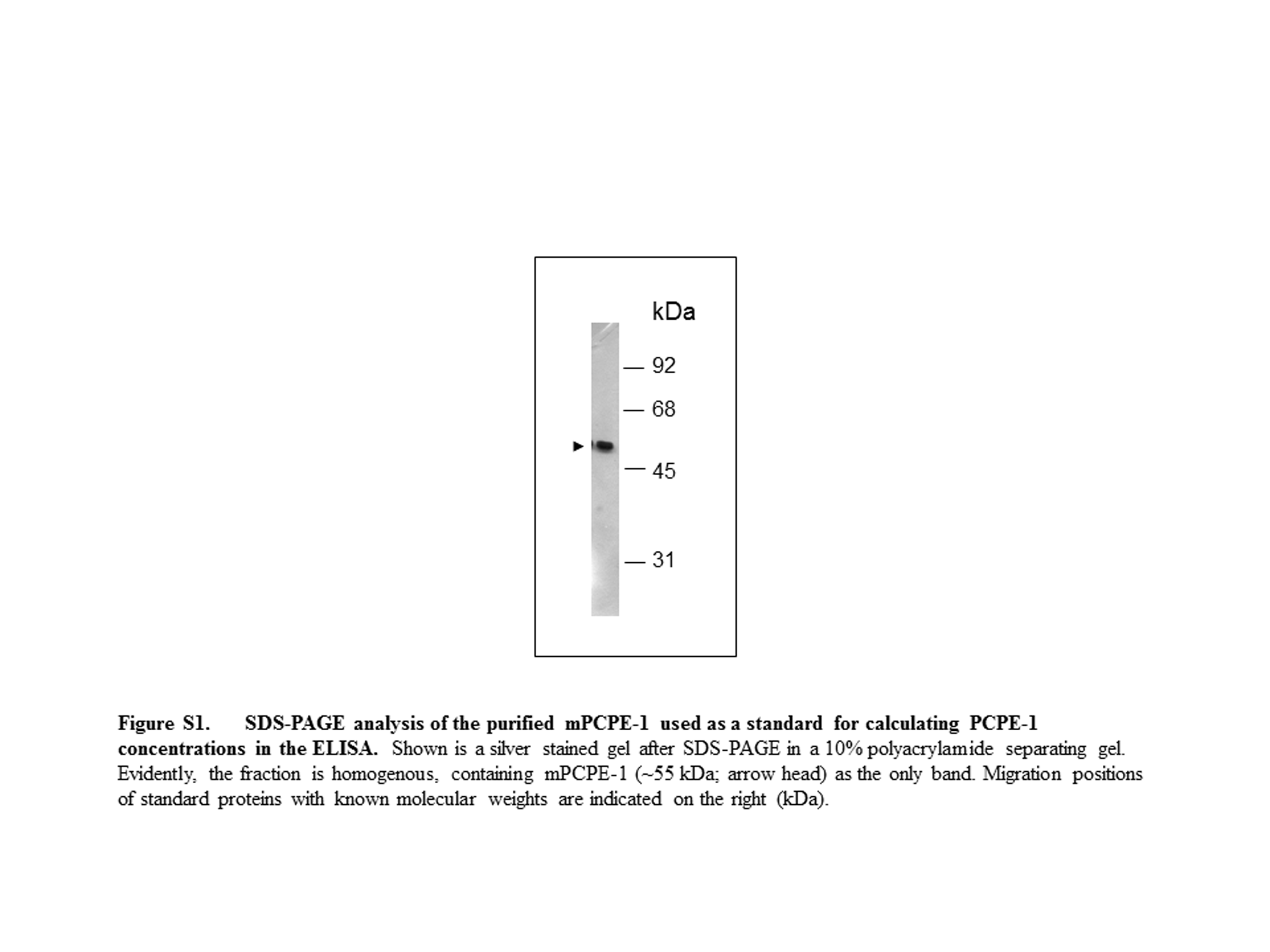

Supplement: S1 Fig — (TIF) [file pone.0159606.s001.tif]
