# Supplementary material for: Procollagen C-Proteinase Enhancer 1 (PCPE-1) as a Plasma Marker of Muscle and Liver Fibrosis in Mice
Source: PLoS One. 2016 Jul 26;11(7):e0159606. doi: 10.1371/journal.pone.0159606 (PMC4961444; doi:10.1371/journal.pone.0159606)
Supplement: S1 Table — (DOCX) [file pone.0159606.s002.docx]

**Table 1S. Determination of inter-assay coefficient of variability for the mPCPE-1 sandwich ELISA**

| **PCPE-1 plasma concentration (ng/ml)** | | | | | |
| --- | --- | --- | --- | --- | --- |
| **Mean ± SD** | **1:40** | | **1:20** | | **Dilution** |
|  | **result 2** | **result 1** | **result 2** | **result 1** | **Assay No.** |
| 218.17 ± 7.25 | 225.12 | 225.72 | 210.70 | 211.14 | 1 |
| 212.78 ± 19.09 | 229.59 | 234.03 | 193.73 | 193.78 | 2 |
| 198.70 ± 11.94 | 213.83 | 203.99 | 195.74 | 181.24 | 3 |
| 205.79 ± 14.84 | 222.15 | 218.28 | 195.90 | 186.82 | 4 |
| 187.37 ± 4.03 | 192.62 | 189.03 | 181.60 | 186.22 | 5 |
| 190.05 ± 10.47 | 178.83 | 180.37 | 201.19 | 199.80 | 6 |
| 180.61 ± 9.99 | 188.49 | 192.14 | 173.74 | 168.08 | 7 |
| 188.74 ± 6.07 | 183.27 | 183.27 | 197.94 | 190.50 | 8 |
| 195.45 ± 5.82 | 200.68 | 200.61 | 193.95 | 186.54 | 9 |
| 209.95 ± 13.73 | 229.69 | 215.11 | 201.20 | 193.79 | 10 |
| **198.76** |  | | | | **Mean of means** |
| **12.45** |  | | | | **SD of means** |
| **6.26** |  | | | | **%CV** |

PCPE-1 concentration in a plasma sample from a six weeks old C57/BL/6 (wild type) mouse was determined in 10 independent experiments performed on different days and calculated based on a calibration curve performed on the same day. The plasma sample was diluted 1:20 and 1:40 and PCPE-1 concentration for each dilution was determined in duplicates.
