# Supplementary material for: Procollagen C-Proteinase Enhancer 1 (PCPE-1) as a Plasma Marker of Muscle and Liver Fibrosis in Mice
Source: PLoS One. 2016 Jul 26;11(7):e0159606. doi: 10.1371/journal.pone.0159606 (PMC4961444; doi:10.1371/journal.pone.0159606)
Supplement: S2 Table — (DOCX) [file pone.0159606.s003.docx]

**Table S2. Determination of intra-assay coefficient of variability for the mPCPE-1 sandwich ELISA – the *mdx* model**

| **PCPE-1 plasma concentration (ng/ml)** | | | | | | | | | |
| --- | --- | --- | --- | --- | --- | --- | --- | --- | --- |
| **Dilution** | **1:20** | | | **1:40** | | | **mean** | **SD** | **%CV** |
| **Sample** | **result 1** | **result 2** | **result 3** | **result 1** | **result 2** | **result 3** |  |  |  |
| **1** | 256.46 | 244.64 | 238.94 | 276.59 | 277.82 | 268.40 | 260.48 | 15.02 | **5.77** |
| **2** | 191.19 | 183.88 | 183.69 | 186.49 | 182.69 | 200.64 | 188.10 | 6.27 | **3.33** |
| **3** | 176.71 | 180.61 | 182.93 | 198.04 | 199.08 | 194.79 | 188.69 | 8.90 | **4.71** |
| **4** | 202.26 | 204.68 | 195.76 | 241.29 | 236.54 | 250.71 | 221.87 | 21.55 | **9.71** |
| **5** | 241.74 | 247.81 | 243.91 | 283.29 | 282.91 | 280.87 | 263.42 | 19.03 | **7.23** |
| **6** | 176.70 | 177.03 | 178.35 | 192.39 | 171.62 | 190.08 | 181.03 | 7.54 | **4.17** |
| **7** | 170.18 | 177.03 | 182.54 | 195.30 | 194.17 | 193.62 | 185.47 | 9.59 | **5.17** |
| **8** | 137.50 | 130.99 | 127.06 | 207.93 | 212.57 | 196.09 | 168.69 | 37.29 | **22.11** |
| **9** | 304.17 | 304.69 | 300.71 | 365.64 | 366.52 | 363.69 | 334.24 | 31.08 | **9.30** |
| **10** | 201.50 | 204.16 | 205.34 | 243.91 | 233.29 | 238.18 | 221.06 | 17.70 | **8.01** |
| **11** | 288.42 | 288.91 | 289.48 | 371.56 | 359.10 | 393.47 | 331.82 | 44.05 | **13.27** |
| **12** | 249.21 | 241.94 | 246.24 | 339.09 | 335.08 | 322.41 | 288.99 | 43.54 | **15.07** |
| **13** | 311.80 | 311.61 | 313.34 | 388.12 | 386.23 | 385.92 | 349.50 | 37.27 | **10.66** |
| **14** | 252.56 | 253.25 | 252.04 | 281.23 | 282.28 | 281.64 | 267.17 | 14.56 | **5.45** |
| **15** | 248.07 | 260.83 | 279.36 | 278.79 | 298.59 | 283.04 | 274.78 | 16.23 | **5.91** |
| **16** | 275.30 | 272.24 | 266.95 | 303.40 | 306.17 | 310.84 | 289.15 | 17.95 | **6.21** |
| **Mean of %CV** |  |  |  |  |  |  |  |  | **8.5** |

Plasma samples from four mice from each group (control and CCl_4_-treated, each at four and 8.5 months of age) were diluted 1:20 and 1:40. The PCPE-1 plasma concentrations were determined in triplicates for each dilution. Measurements were performed on the same day and results were calculated based on a calibration curve run in parallel on the same day. Samples 1-4 and 5-8 are from four and 8.5 months old control mice, respectively; samples 9-12 and 13-16 are from four and 8.5 months old *mdx* mice, respectively.
