# Supplementary material for: Procollagen C-Proteinase Enhancer 1 (PCPE-1) as a Plasma Marker of Muscle and Liver Fibrosis in Mice
Source: PLoS One. 2016 Jul 26;11(7):e0159606. doi: 10.1371/journal.pone.0159606 (PMC4961444; doi:10.1371/journal.pone.0159606)
Supplement: S3 Table — (DOCX) [file pone.0159606.s004.docx]

**Table S3. Determination of intra-assay coefficient of variability for the mPCPE-1 sandwich ELISA – the CCl_4_ liver fibrosis model**

| **PCPE-1 plasma concentration (ng/ml)** | | | | | | | |
| --- | --- | --- | --- | --- | --- | --- | --- |
| **Dilution** | **1:20** | | **1:40** | | **mean** | **S.D.** | **%CV** |
| **Mouse No.** | **result 1** | **result 2** | **result 1** | **result 2** |  |  |  |
| **1** | 229.48 | 203.81 | 273.31 | 244.47 | 237.77 | 25.15 | **10.58** |
| **2** | 171.52 | 170.03 | 181.39 | 180.76 | 175.93 | 5.18 | **2.94** |
| **3** | 222.84 | 211.38 | 227.02 | 220.18 | 220.35 | 5.73 | **2.60** |
| **4** | 116.68 | 114.86 | 137.66 | 139.67 | 127.22 | 11.49 | **9.03** |
| **5** | 202.52 | 205.71 | 204.93 | 214.60 | 206.94 | 4.58 | **2.21** |
| **6** | 173.10 | 171.79 | 173.05 | 171.89 | 172.46 | 0.62 | **0.36** |
| **7** | 157.37 | 163.04 | 200.97 | 198.69 | 180.02 | 19.93 | **11.07** |
| **8** | 188.30 | 193.56 | 194.34 | 204.42 | 195.16 | 5.83 | **2.99** |
| **9** | 253.28 | 258.54 | 294.70 | 289.82 | 292.26 | 18.35 | **6.28** |
| **10** | 200.15 | 191.58 | 224.15 | 219.26 | 221.71 | 13.38 | **6.04** |
| **11** | 248.70 | 257.65 | 274.81 | 275.07 | 274.94 | 11.33 | **4.12** |
| **12** | 272.17 | 252.95 | 266.86 | 277.44 | 272.15 | 9.12 | **3.35** |
| **13** | 259.64 | 268.03 | 260.93 | 285.23 | 273.08 | 10.20 | **3.73** |
| **14** | 224.43 | 222.38 | 259.60 | 236.40 | 248.00 | 14.80 | **5.97** |
| **15** | 220.90 | 222.50 | 245.80 | 270.60 | 258.20 | 20.26 | **7.85** |
| **16** | 271.22 | 282.45 | 283.78 | 261.32 | 272.55 | 9.13 | **3.35** |
| **Mean of %CV** |  |  |  |  |  |  | **5.15** |

Plasma samples from eight C57/Bl/6 mice that were given olive oil twice a week for six weeks (control) and eight mice that were treated identically with CCl_4_ were each diluted 1:20 and 1:40 and PCPE-1 concentrations for each dilution were determined in duplicates. Measurements were performed on the same day and results were calculated based on a calibration curve run in parallel on the same day. 1-8, control mice; 9-16, CCl_4_-treated mice.
